# Prepubertal Serum Concentrations of Organochlorine Pesticides and Age at Sexual Maturity in Russian Boys

**DOI:** 10.1289/ehp.1409022

**Published:** 2015-05-22

**Authors:** Thuy Lam, Paige L. Williams, Mary M. Lee, Susan A. Korrick, Linda S. Birnbaum, Jane S. Burns, Oleg Sergeyev, Boris Revich, Larisa M. Altshul, Donald G. Patterson, Russ Hauser

**Affiliations:** 1 Environmental and Occupational Medicine and Epidemiology Program, Department of Environmental Health, Harvard T.H. Chan School of Public Health, Boston, Massachusetts, USA; 2 Gradient, Cambridge, Massachusetts, USA; 3 Department of Biostatistics, and; 4 Department of Epidemiology, Harvard T.H. Chan School of Public Health, Boston, Massachusetts, USA; 5 Pediatric Endocrine Division, Department of Pediatrics, and; 6 Department of Cell and Developmental Biology, University of Massachusetts Medical School, Worcester, Massachusetts, USA; 7 Channing Division of Network Medicine, Department of Medicine, Brigham and Women’s Hospital, Harvard Medical School, Boston, Massachusetts, USA; 8 National Cancer Institute, National Institutes of Health, Department of Health and Human Services, Research Triangle Park, North Carolina, USA; 9 Department of Genomics and Human Genetics, Vavilov Institute of General Genetics, Russian Academy of Sciences, Moscow, Russia; 10 Chapaevsk Medical Association, Chapaevsk, Samara Region, Russia; 11 Institute for Forecasting, Russian Academy of Sciences, Moscow, Russia; 12 Exposure, Epidemiology, and Risk Program, Department of Environmental Health, Harvard T.H. Chan, School of Public Health, Boston, Massachusetts, USA; 13 Environmental Health and Engineering Inc., Needham, Massachusetts, USA; 14 EnviroSolutions Consulting Inc., Auburn, Georgia, USA; 15 Axys Analytical Solutions, Sidney, British Columbia, Canada; 16 Exponent Inc., Maynard, Massachusetts, USA

## Abstract

**Background:**

Few human studies have evaluated the impact of childhood exposure to organochlorine pesticides (OCP) on pubertal development.

**Objective:**

We evaluated associations of serum OCP concentrations [hexachlorobenzene (HCB), β-hexachlorocyclohexane (βHCH), and *p,p*-dichlorodiphenyldichloroethylene (*p,p*´-DDE)] with age at attainment of sexual maturity among boys.

**Methods:**

From 2003 through 2005, 350 8- to 9-year-old boys from Chapaevsk, Russia, with measured OCPs were enrolled and followed annually for 8 years. We used multivariable interval-censored models to evaluate associations of OCPs (quartiles) with three physician-assessed measures of sexual maturity: Tanner stage 5 for genitalia growth, Tanner stage 5 for pubic hair growth, or testicular volume (TV) ≥ 20 mL in either testis.

**Results:**

In adjusted models, boys with higher HCB concentrations achieved sexual maturity reflected by TV ≥ 20 mL a mean of 3.1 months (95% CI: –1.7, 7.8), 5.3 months (95% CI: 0.6, 10.1), and 5.0 months (95% CI: 0.2, 9.8) later for quartiles Q2, Q3, and Q4, respectively, compared with Q1 (*p* trend = 0.04). Tanner stage 5 for genitalia growth was attained a mean of 2.2 months (95% CI: –3.1, 7.5), 5.7 months (95% CI: 0.4, 11.0), and 3.7 months (95% CI: –1.7, 9.1) later for quartiles Q2, Q3, and Q4, respectively, of βHCH compared with Q1 (*p* trend = 0.09). Tanner stage 5 for pubic hair growth occurred 6–9 months later on average for boys in the highest versus lowest quartile for HCB (*p* trend < 0.001), βHCH (trend *p* = 0.01), and *p,p*´-DDE (*p* trend = 0.04). No associations were observed between *p,p*´-DDE and Tanner stage 5 for genitalia growth or TV ≥ 20 mL.

**Conclusions and relevance:**

Higher prepubertal serum HCB and βHCH concentrations were associated with a later age at attainment of sexual maturity. Only the highest quartile of serum *p,p*´-DDE was associated with later pubic hair maturation.

**Citation:**

Lam T, Williams PL, Lee MM, Korrick SA, Birnbaum LS, Burns JS, Sergeyev O, Revich B, Altshul LM, Patterson DG Jr, Hauser R. 2015. Prepubertal serum concentrations of organochlorine pesticides and age at sexual maturity in Russian boys. Environ Health Perspect 123:1216–1221; http://dx.doi.org/10.1289/ehp.1409022

## Introduction

Organochlorine pesticides (OCPs) such as hexachlorobenzene (HCB), β-hexachlorocyclohexane (βHCH), and 1,1,1,-trichloro-2,2,bis(*p*-chlorophenyl)ethane (DDT) were used as insecticides and fungicides for decades until the 1980s (Barber et al. 2005; Jaga and Dharmani 2003; Jung et al. 1997). Though production of these pesticides has been banned in most countries (Barber et al. 2005; Breivick et al. 1999; Jaga and Dharmani 2003), DDT is still used to control malaria (Jaga and Dharmani 2003), and HCB and βHCH are unintentional by-products from manufacturing of other chlorinated chemicals (Courtney 1979; Jung et al. 1997). The lipophilic and persistent nature of these environmentally stable compounds and their ability to biomagnify through the food chain (Barber et al. 2005; Jaga and Dharmani 2003; Jung et al. 1997) are primary reasons for ongoing exposure in the general population. These OCPs and their metabolites such as *p,p*´-DDE (dichlorodiphenyldichloroethylene) are endocrine-disrupting chemicals (EDCs) that affect puberty and reproductive development in rodents (Courtney 1979; Gray et al. 2001; Kelce et al. 1995; Van Velsen et al. 1986).

Puberty is a complex process characterized by physical and hormonal changes regulated by two parallel but independent processes: adrenal maturation (adrenarche) and the maturation of the hypothalamic–pituitary–gonadal (HPG) axis (Havelock et al. 2004; Kronenberg et al. 2008). In boys, virilization of the genitalia and testicular enlargement are cues of HPG activation, whereas pubic hair growth is often associated with adrenarche (Havelock et al. 2004). Early attainment of male sexual maturity is associated with a variety of adverse effects including antisocial behaviors, short adult height, reduced fertility, and prostate and testicular cancer (Golub et al. 2008; Patton and Viner 2007). Later male maturity has been linked to poor body image, depression, and osteoporosis (Golub et al. 2008; Patton and Viner 2007; Rosen and Foster 2001). Factors that may affect the timing of sexual maturation include deficits in energy/micronutrients and possibly environmental chemicals such as OCPs (Golub et al. 2008; Kaplowitz 2008; Mouritsen et al. 2010; Mustanski et al. 2004; Rogol et al. 2000).

Although rodent studies have demonstrated that fetal and postnatal exposure to *p,p*´-DDE, DDT’s primary metabolite, delayed male preputial separation (Kelce et al. 1995), a marker of male puberty, none have reported on associations with sexual maturation. Sexual maturity in rodents is assessed by sperm production or mating behavior. The age at production of mature sperm varies widely among strains, with estimates ranging from postnatal day 40 to 100; therefore, assessing the timing of sexual maturation in rodents is imprecise (Campion et al. 2013; Marty et al. 2003). HCB, βHCH, and *p,p*´-DDE have been associated with reproductive and developmental abnormalities in male rodent offspring, including fetal growth retardation, delayed testicular descent, and reduced fertility (Courtney 1979; Gray et al. 2001; Kelce et al. 1995; Quinn et al. 2008; Simon et al. 1979; Van Velsen et al. 1986). Epidemiologic evidence on the association of OCPs with pubertal development is limited, and the direction of the findings is inconsistent (Den Hond et al. 2011). In a cross-sectional study of Flemish boys 14–15 years of age, higher serum levels of HCB and *p,p*´-DDE were associated with earlier genital and pubic hair development (Den Hond et al. 2011). In contrast, among a cohort of Russian boys residing in an environmentally contaminated town, we reported that higher serum HCB concentrations were associated with later pubertal onset (Lam et al. 2014). In the present analysis, we investigated the association of prepubertal serum concentrations of HCB, βHCH, and *p,p*´-DDE with age at male sexual maturity in the same cohort of Russian boys.

## Methods

*Study population*. The Russian Children’s Study is a prospective cohort study of 499 boys, enrolled at age 8–9 years in 2003–2005, residing in Chapaevsk, Russia, a community contaminated with organochlorine compounds, including OCPs (Burns et al. 2009; Lam et al. 2013). Exclusion criteria included severe chronic medical conditions or institutionalization. OCP concentrations were not measured for the first 144 boys enrolled because the study initially focused on dioxins, and 5 additional boys were excluded due to chronic illnesses impacting growth, leaving 350 boys for the present analysis. The study was approved by the Human Studies Institutional Review Boards of the Chapaevsk Medical Association, Harvard T.H. Chan School of Public Health, University of Massachusetts Medical School, and Brigham and Women’s Hospital. The parent/guardian gave informed consent and the boys signed assent forms before participation.

At study entry, the parent/guardian completed nurse-administered health and lifestyle questionnaires on demographics, medical and family history, household smoking, breastfeeding of the child in the study, household income, and parental education. At the same visit, the parent/guardian completed a Russian Institute of Nutrition food frequency questionnaire to ascertain the boy’s usual dietary intake. Birth outcomes (e.g., birth weight, gestational age) were abstracted from the medical records. Blood lead levels (BLLs) were measured from the boys’ blood samples collected at enrollment (ages 8–9 years) (Hauser et al. 2008; Williams et al. 2010).

*Physical examination and pubertal assessment*. A standardized physical examination was performed at study entry and annually for up to eight follow-up visits by a single physician (O.S.). Testicular volume (TV) was measured by palpation and comparison to a Prader orchidometer. Pubertal assessments were also performed by visual inspection using established Tanner stage criteria for genitalia and pubic hair on a scale of 1 (immature) to 5 (mature) (Tanner and Whitehouse 1976). Sexual maturity was defined as Tanner stage 5 for genitalia growth, or Tanner stage 5 for pubic hair growth, or TV ≥ 20 mL for either testis (Basu 2011).

*Organochlorine pesticide exposure assessment*. At study entry, fasting blood samples were collected from participants, and serum aliquots were stored at –35°C until shipment on dry ice to the U.S. Centers for Disease Control and Prevention, Atlanta, Georgia, for analysis. The samples, including method blanks and quality control samples, were spiked with ^13^C_12_-labeled pesticides, extracted by a C18 solid-phase extraction followed by a multi-column automated cleanup and enrichment procedure (Sjödin et al. 2004; Turner et al. 1997). Samples were analyzed with high-resolution mass spectrometry in selective ion monitoring mode (Barr et al. 2003). Total serum lipid content of the aliquot was determined from enzymatic measurements of total cholesterol and triglycerides (Phillips et al. 1989). The analytical coefficients of variation for individual OCPs in quality control/quality assurance samples ranged between 10% and 15%. All OCP concentrations were expressed on a wet-weight basis (picograms per gram serum) or on a lipid-normalized basis (nanograms per gram lipid) (division of wet-weight levels by lipid concentrations).

*Statistical analysis*. Unadjusted and adjusted interval-censored survival analyses were used to evaluate the associations between boys’ serum OCP concentrations (in quartiles) and age at sexual maturity; the three higher quartiles were each compared with the lowest quartile, and tests for trend were performed by modeling OCP quartiles as an ordinal variable. A normal distribution for age at sexual maturity was assumed. Use of an interval-censored model allows for the fact that sexual maturity may occur in the interval between study visits (interval-censored), or may not yet have occurred by the last study visit (right-censored). We calculated the overall mean age of sexual maturity for each maturity measure, and the mean age of maturity for each OCP quartile assuming the mean or reference levels for other model covariates.

Covariates considered in the models included *a priori* identified potential predictors of sexual maturity at baseline (Table 1): maternal age at son’s birth, household tobacco use, boys’ birth weight and gestational age, breastfeeding duration, diet, and BLLs at study enrollment, as well as socioeconomic status (SES) indicators (e.g., biological father’s absence from the household, household income, parental education). A core model was developed first by evaluating the associations of each covariate with sexual maturity and retaining those with a *p* < 0.20. Covariates meeting this criterion were then included in a full model and backwards selection (likelihood ratio test) was used to exclude covariates with *p* > 0.10. To check for confounding, covariates were added individually back into the final model and retained if they resulted in a ≥ 10% change in the OCP coefficient estimates obtained from the trend test. Separate core models were developed for each maturity measure. Because OCPs are lipophilic and because of the potential for bias, rather than modeling lipid-normalized OCPs, we instead chose to use the wet weights for OCPs and adjust for concurrently measured serum total lipids by including this as a covariate in the model (Li et al. 2013; Schisterman et al. 2005). However, we also performed an alternative analysis using quartiles of lipid-normalized serum OCP concentrations rather than wet-weight serum OCPs. Statistical significance was defined as *p* ≤ 0.05. All statistical analyses were conducted using SAS statistical software, version 9.2 (SAS Institute Inc., Cary, NC).

**Table 1:** Characteristics of participants in the Russian Children’s Study with serum organochlorine pesticide measurements at enrollment (ages 8–9 years) [mean ± SD or *n* (%)].

Characteristic	Total boys (*n *= 350)
Child characteristics
Growth measurements
Height (cm)	129.0 ± 6
Weight (kg)	26.6 ± 5.3
BMI	15.9 ± 2.3
WHO height *z*-score	0.12 ± 1.0
WHO BMI *z*-score	–0.17 ± 1.3
Birth and neonatal history
Birth weight (kg)	3.3 ± 0.5
Gestational age (weeks)	39.0 ± 1.8
Preterm birth (gestational age < 37 weeks)	33 (9)
Macronutrients
Total calories (calories)	2695.7 ± 931.0
Percent carbohydrates	54.3 ± 6.6
Percent fat	34.2 ± 5.9
Percent protein	11.6 ± 1.6
Other characteristics
Blood lead levels ≥ 5 μg/dL	86 (25)
Parental and residential characteristics
Any household smoking during pregnancy	58 (17)
Maternal age at son’s birth (< 25 years)	222 (63)
Maternal age at menarche (years)	13.3 ± 1.3
Biological father absent from household	123 (35)
Maximum parental education
High school or less	29 (8)
Junior college/technical school	198 (57)
University/postgraduate training	121 (35)
Household income (US$ per month)
< 175	107 (31)
175–250	88 (25)
> 250	154 (44)
BMI, body mass index. Percentages may not total 100% due to rounding. Missing: birth weight, *n *= 1; household smoking during pregnancy, *n *= 5; maternal age at son’s birth, *n *= 3; maternal age at menarche, *n *= 26; parental education, *n *= 2; household income, *n *= 1; macronutrients, *n *= 3.	

Prior analyses in this cohort have found OCPs to be associated with reduced body mass index (BMI) and height *z*-scores [defined according to World Health Organization (WHO) child growth standards] (Burns et al. 2012; de Onis et al. 2007): These markers of growth are, in turn, strongly associated with age at sexual maturity and thus may be on the causal pathway between OCPs and sexual maturity. Because of these previously identified relationships, BMI and height *z*-scores were excluded from the primary analysis, but sensitivity analyses were conducted to evaluate these mediators by adding them to the final models. Sensitivity analyses were also conducted to assess robustness of findings with further adjustment for maternal age at menarche (unavailable for 8% of participants).

## Results

*Exposure and demographic characteristics*. Median (25th, 75th percentiles) concentrations for wet-weight serum HCB, βHCH, and *p,p*´-DDE were 754 (522, 1,159), 814 (560, 1,294), and 1,408 (904, 2,324) pg/g serum, respectively. The median (25th, 75th percentiles) concentrations for lipid-normalized serum HCB, βHCH, and *p,p*´-DDE were 159 (107, 247), 68 (114, 272), and 287 (189, 492) ng/g lipid, respectively. No values were below the limit of detection. Most boys had BMI and height *z*-scores within 1 SD of the WHO mean (Table 1). Boys with and without serum OCP measurements (*n* = 350 vs. 144) did not differ significantly by BMI, height *z*-scores, or birth characteristics at the 0.05 significance level (Lam et al. 2013). However, more boys with OCP measurements were in the highest parental education categories and household income categories than were those without. Spearman correlations between the OCPs were *r* = 0.34 for HCB and *p,p*´-DDE, *r* = 0.54 for βHCH and HCB, and *r* = 0.61 for βHCH and *p,p*´-DDE.

*Sexual maturity characteristics*. Among the 72% of boys who were followed until at least the 16- to 17-year-old study visit, 94%, 87%, and 44% had attained TV ≥ 20 mL, Tanner stage 5 for genitalia growth, and Tanner stage 5 for pubic hair growth, respectively. The overall estimated mean age [95% confidence interval (CI)] of sexual maturity for TV ≥ 20 mL, Tanner stage 5 for genitalia growth, and Tanner stage 5 for pubic hair growth was 13.8 (13.7, 14.0), 14.7 (14.6, 14.9), and 16.0 years (15.8, 16.2), respectively.

**Figure 1:**
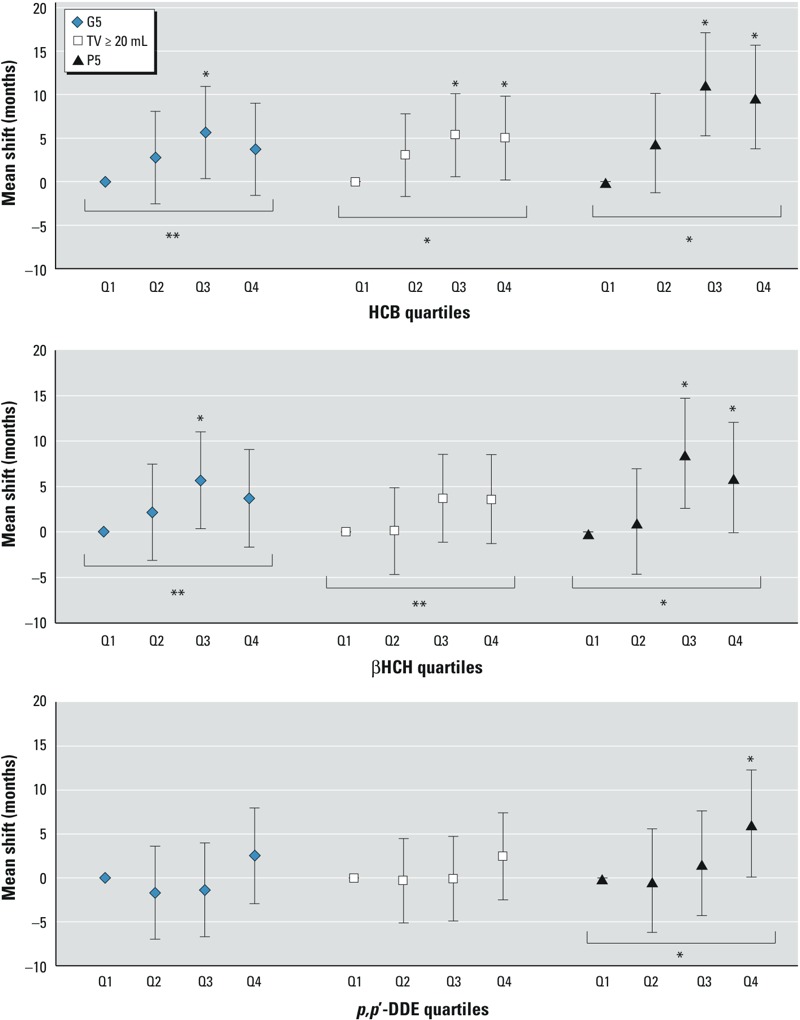
Adjusted mean shifts in age at sexual maturity (months, 95% CIs) by quartiles of wet-weight serum OCP concentrations among 350 Russian boys, relative to the lowest quartile (Q1). Baseline covariates for each model are as follows: G5: boy’s total serum lipids, macronutrients (total caloric intake, percent calories from dietary carbohydrates, fat, protein) (missing macronutrients, *n *= 3); TV ≥ 20 mL: boys’ total serum lipids, birth weight, blood lead levels, biological father’s absence from the household (missing birth weight, *n *= 1); P5: boys’ total serum lipids, biological father’s absence from the household. HCB wet-weight quartiles (pg/g serum): Q1, 169–516; Q2, 517–751; Q3, 752–1,156; Q4, 1,157–15,482. βHCH wet-weight quartiles (pg/g serum): Q1, 209–567; Q2, 568–814; Q3, 815–1,294; Q4, 1,295–13,732. *p,p*´-DDE wet-weight quartiles (pg/g serum): Q1, 261–907; Q2, 908–1,406; Q3, 1,407–2,327; Q4, 2,328–41,301. *p ≤ 0.05. **p ≤ 0.10.

*Associations of serum OCPs with TV ≥ 20 mL and Tanner stage 5 for genitalia growth*. The multivariable model for TV ≥ 20 mL included the covariates total serum lipids, biological father’s absence from the household, boy’s birth weight, and boy’s BLLs ≥ 5 μg/dL. For Tanner stage 5 for genitalia growth, the multivariable model included the covariates total serum lipids and macronutrients (i.e., total caloric intake, percent calories from carbohydrates, fat, and protein). Higher serum HCB concentrations were associated with later attainment of both TV ≥ 20 mL and Tanner stage 5 for genitalia. HCB quartiles 3 and 4 were associated with approximately 5 months later TV ≥ 20 mL compared with the lowest quartile, with a significant trend. However, only quartile 3 of HCB was associated with later attainment for Tanner stage 5 for genitalia (by 5.6 months, 95% CI: 0.3, 10.9), with attenuation in quartile 4 and no significant trend. A similar pattern was observed for βHCH and Tanner stage 5 for genitalia growth, with a significantly later age at maturity only observed within the third quartile; no association was observed between βHCH and reaching TV ≥ 20 mL (Table 2, Figure 1). The estimated mean ages at maturity ranged from 13.4 to 13.8 years for TV ≥ 20 mL and from 14.5 to 14.8 years for attaining Tanner stage 5 for genitalia. We observed no statistically significant association for *p,p*´-DDE with later TV or genital maturity.

**Table 2:** Adjusted mean shifts in age at sexual maturity [months (95% CIs)] by quartiles of wet-weight serum OCP concentrations among 350 Russian boys.

Serum OCP quartile	G5 (*n *= 347)^*a*^		TV ≥ 20 mL (*n *= 349)^*b*^		P5 (*n *= 350)^*c*^	
	Mean shift	*p*-Value	Mean shift	*p*-Value	Mean shift	*p*-Value
HCB^*d*^
Q1 (low)	Reference		Reference		Reference
Q2	2.78 (–2.53, 8.09)	0.31	3.05 (–1.72, 7.81)	0.21	4.43 (–1.28, 10.14)	0.13
Q3	5.64 (0.34, 10.94)	0.04	5.34 (0.57, 10.10)	0.03	11.20 (5.27, 17.13)	< 0.001
Q4 (high)	3.71 (–1.59, 9.00)	0.17	5.01 (0.21, 9.82)	0.04	9.73 (3.78, 15.67)	0.001
*p for trend*		0.10		0.02		< 0.001
βHCH^*e*^
Q1 (low)	Reference		Reference		Reference
Q2	2.18 (–3.12, 7.48)	0.42	0.10 (–4.68, 4.88)	0.97	1.17 (–4.63, 6.96)	0.69
Q3	5.69 (0.36, 11.02)	0.04	3.71 (–1.13, 8.54)	0.13	8.67 (2.61, 14.74)	0.01
Q4 (high)	3.71 (–1.65, 9.08)	0.17	3.63 (–1.27, 8.53)	0.15	5.99 (–0.08, 12.07)	0.05
*p for trend*		0.09		0.07		0.01
*p,p*´-DDE^*f*^
Q1 (low)	Reference		Reference		Reference
Q2	–1.68 (–6.98, 3.61)	0.53	–0.32 (–5.10, 4.47)	0.90	–0.30 (–6.19, 5.59)	0.92
Q3	–1.34 (–6.67, 3.99)	0.62	–0.09 (–4.89, 4.71)	0.97	1.67 (–4.29, 7.63)	0.58
Q4 (high)	2.52 (–2.92, 7.97)	0.36	2.45 (–2.49, 7.39)	0.33	6.19 (0.11, 12.27)	0.05
*p* for trend		0.37		0.35		0.04
Abbreviations: G5, Tanner stage 5 for genitalia growth; P5, Tanner stage 5 for pubic hair growth. ^***a***^G5 model adjusted for baseline covariates: boys’ total serum lipids, macronutrients (total caloric intake, percent calories from dietary carbohydrates, fat, and protein); missing macronutrients, *n *= 3. ^***b***^TV ≥ 20 mL model adjusted for baseline covariates: boys’ total serum lipids, birth weight, blood lead levels, biological father’s absence from the household; missing birth weight, *n *= 1. ^***c***^P5 model adjusted for baseline covariates: boys’ total serum lipids, biological father’s absence from the household. ^***d***^HCB wet-weight quartiles (Q1–Q4, pg/g serum): Q1, 169–516; Q2, 517–751; Q3, 752–1,156; Q4, 1,157–15,482. ^***e***^βHCH wet-weight quartiles (Q1–Q4, pg/g serum): Q1, 209–567; Q2, 568–814; Q3, 815–1,294; Q4, 1,295–13,732. ^***f***^*p,p*´-DDE wet-weight quartiles (Q1–Q4, pg/g serum): Q1, 261–907; Q2, 908–1,406; Q3, 1,407–2,327; Q4, 2,328–41,301.						

*Associations of serum OCPs with Tanner stage 5 for pubic hair growth*. The models for age at attainment of Tanner stage 5 for pubic hair growth were adjusted for total serum lipids and biological father’s absence from the household. In adjusted models, boys with higher HCB and βHCH concentrations (Q3 and Q4) attained Tanner stage 5 for pubic hair growth > 6 months later, on average, than those in the lowest quartile, although the associations were attenuated in the highest quartile (HCB trend *p* < 0.001; βHCH trend *p* = 0.01; Table 2, Figure 1). Adjusted mean ages at attainment of Tanner stage 5 for pubic hair growth ranged from 15.2 to 16.1 years over HCB quartiles and 15.4 to 15.9 years over βHCH quartiles. The association of *p,p*´-DDE with later Tanner stage 5 for pubic hair growth (trend *p* = 0.04) was primarily driven by quartile 4 (Figure 1).

*Sensitivity analyses*. In sensitivity analyses adjusted for maternal age at menarche, the associations of TV ≥ 20 mL and Tanner stage 5 for genitalia growth with HCB and βHCH were consistent with primary models (see Supplemental Material, Table S1). Adjustment of primary models for baseline BMI and height *z*-scores resulted in slight attenuation of the associations of HCB and βHCH with TV ≥ 20 mL and Tanner stage 5 for genitalia growth. In contrast, after adjustment for BMI and height *z*-scores, βHCH and *p,p*´-DDE were no longer associated with Tanner stage 5 for pubic hair growth, whereas the association of HCB with Tanner stage 5 for pubic hair growth was attenuated but remained significant.

*Associations of lipid-normalized serum OCPs with sexual maturity*. Analyses modeling lipid-normalized serum OCPs yielded stronger associations of βHCH with Tanner stage 5 for pubic hair growth, TV ≥ 20 mL, and Tanner stage 5 for genitalia growth, compared to wet-weight models adjusted for serum lipids, but primary conclusions were unaffected (see Supplemental Material, Table S2 and Figure S1). Lipid-normalized serum HCB associations with sexual maturity were attenuated compared with the wet-weight models; the association with TV ≥ 20 mL became nonsignificant. Models using lipid-normalized *p,p*´-DDE demonstrated a stronger association in comparison with wet-weight models for genitalia and TV ≥ 20 mL, although the association with pubic hair was attenuated. Additional analyses with lipid-normalized OCP measures further adjusted for maternal age at menarche and BMI and height *z*-scores did not substantially change our results (see Supplemental Material, Table S3).

*Associations of OCP mixtures with sexual maturity.* Estimated associations of age at maturity with either HCB or βHCH were very similar after additional adjustment for *p,p´-DDE.* However, in models including both HCB and βHCH, associations for HCB were attenuated and remained significant only for Tanner stage 5 for pubic hair growth; associations for βHCH were markedly attenuated for all maturity markers, and none approached significance. There were no associations of *p,p*´-DDE with Tanner stage 5 for pubic hair growth in models of multiple OCPs (see Supplemental Material, Table S4).

## Discussion

In our longitudinal study, we found associations of higher prepubertal serum HCB, βHCH, and *p,p*´-DDE concentrations with later sexual maturity defined as Tanner stage 5 for pubic hair growth, as well as an association of higher HCB with later attainment of TV ≥ 20 mL. Our recent analysis of this Russian cohort found later pubertal onset among boys with higher serum HCB concentrations (Lam et al. 2014). These pubertal onset findings, along with the results of our current analysis on the association of HCB with later sexual maturity, suggests that there is, on average, a similar 5-month delay in both pubertal onset and attainment of sexual maturation. Thus, on average, the pace (tempo between onset and sexual maturity) of puberty did not change in relation to HCB exposure.

Few epidemiologic studies have assessed the association of OCPs with age at sexual maturity and the findings have been inconsistent, possibly due to differences in study design, definition of maturity, exposure mixtures, and timing of exposure and outcome assessment (Den Hond et al. 2011; Gladen et al. 2000). A prospective cohort study in North Carolina found no association between lactational or prenatal *p,p*´-DDE exposures and self-reported Tanner genitalia stages in 278 boys 10–15 years of age (Gladen et al. 2000). The North Carolina study differed from ours in assessing gestational exposures and used self-reported Tanner staging, whereas we focused on prepubertal exposures and used physician-assessed staging including gonadal palpation and comparison with an orchidometer, more precise measures of gonadal development (Euling et al. 2008).

In a cross-sectional study of 887 Flemish boys 14–15 years of age living in an urban industrial area, boys with higher HCB levels attained maturity (Tanner stage 3+ for genitalia and pubic hair growth) significantly earlier (Den Hond et al. 2011). Unlike our longitudinal study in which OCPs were measured on average about 8 years before sexual maturation, the Flemish study was cross-sectional (OCPs and puberty were assessed at the same time). Also, the Flemish study defined maturity as Tanner stage 3+ for genitalia and pubic hair growth, which is considered mid-puberty, whereas we defined maturity using Tanner stage 5 for genitalia, Tanner stage 5 for pubic hair growth, or TV ≥ 20 mL. Discordant findings may also reflect different mixtures of industrial exposures in the two populations, and/or differences in serum concentration between the two cohorts; for example, average OCP concentrations in the Russian cohort were much higher than in the Flemish boys (Russian vs. Flemish: HCB median of 158.5 vs. 22.8 ng/g lipid; *p,p´-*DDE median of 286.5 vs. 104 ng/g lipid) (Den Hond et al. 2011; Lam et al. 2013).

Because analyses using lipid-normalized measures rather than wet-weight measures adjusted for total serum lipids did not substantially change our findings, we focused on the wet-weight concentrations because lipid-normalization may introduce some bias into the estimates in some instances (Li et al. 2013; Schisterman et al. 2005). In analyses additionally adjusted for BMI and height *z*-scores, the associations were attenuated, but the overall interpretation did not change for the associations of HCB and βHCH with TV ≥ 20 mL and Tanner stage 5 for genitalia growth. However, higher serum βHCH and *p,p*´-DDE concentrations (Q4) were no longer associated with Tanner stage 5 for pubic hair growth after adjustment for these growth measures (see Supplemental Material, Table S1). This demonstrates the complex interrelationships between puberty and BMI and height, which may be on the causal pathway between OCPs and sexual maturity.

Furthermore, because these OCPs are moderately correlated, we also constructed models including two or three OCPs in the same model to evaluate the impact on associations. Most previously observed associations were attenuated when more than one OCP was included in a model. However, consistent with the robustness of HCB as a predictor of pubertal onset (Lam et al. 2014), and the apparent sensitivity of pubic hair maturation to OCP exposures in this analysis, associations of HCB with genital maturation (TV ≥ 20 mL) and Tanner stage 5 for pubic hair growth remained, with the latter retaining statistical significance even after adjustment for βHCH and/or *p,p*´-DDE.

Although masculinization of the genitalia and testicular growth are regulated by the HPG axis, there are subtle differences at the level of the testes. For instance, testicular growth during puberty primarily reflects spermatogenesis. This is driven by follicle-stimulating hormone from the pituitary, which is stimulated by gonadotropin-releasing hormone from the hypothalamus, in combination with testosterone (Kronenberg et al. 2008; Zawatski and Lee 2013). This process promotes the maturation of the seminiferous tubules and spermatogenesis. Virilization of genitalia is mediated by testosterone, which is produced by the Leydig cells under stimulation of luteinizing hormone from the pituitary (Kronenberg et al. 2008; Zawatski and Lee 2013). In contrast to our previous finding in this cohort of an association of higher concentrations of HCB with later pubertal onset based on testicular volume (but not genitalia), in the maturation analysis we found an association with later maturation for both genitalia development and testicular growth. These data suggest that as puberty advances, HCB may interfere with both the maturation of the seminiferous tubules and spermatogenesis as well as the interstitial Leydig cells (Kronenberg et al. 2008; Zawatski and Lee 2013).

βHCH is hypothesized to have estrogenic action based on evidence of finding testicular atrophy and nephrocalcinosis (typically seen only in females) in exposed male rodents (Van Velsen et al. 1986). βHCH mimics the effects of estradiol without being an agonist for the estrogen receptor (ER), and activates the transcription of promoters containing ERs by an unknown mechanism (Massaad et al. 2002). HCB and *p,p*´-DDE disrupt androgen production and androgen receptor (AR) binding in animals (Hahn et al. 1989; Kelce et al. 1995; Ralph et al. 2003); however, it is unclear how βHCH may affect AR activity. With the potential concentration of organochlorine pesticides in fat and androgen-producing endocrine glands (Foster et al. 1993; Schaefer et al. 2000), perhaps androgen production or androgen metabolism are affected, which could then impair sexual hair development (Randall 2008). These compounds could also be affecting AR binding at the site of action (Randall 2008). Though regulation of adrenarche is not well understood, we hypothesize that HCB may disrupt the production of sex steroids in the zona reticularis of the adrenals and impair activation or responsiveness to androgens at the tissue levels (Havelock et al. 2004; Zawatski and Lee 2013). Obtaining adrenal androgen measurements in our cohort will help elucidate the mechanism for the delay in pubarche.

A limitation of our study is that only a single serum measurement of OCPs was obtained at enrollment. However, OCP measurements were obtained at a sensitive peripubertal exposure window (Lemasters et al. 2000; Pryor et al. 2000). We are also limited in our ability to generalize HCB and βHCH findings to populations with lower exposures. Serum HCB and βHCH concentrations in these boys were among the highest observed among contemporary pediatric populations (Lam et al. 2013), with the 25th percentile for HCB almost eight times the median value of U.S children (Patterson et al. 2009). Therefore, our reference category included boys with relatively high concentrations (i.e., HCB). It is well understood that dose–response relationships for EDCs may be nonlinear (Birnbaum 2012); thus we reported our results in quartiles as a conservative approach so that potential nonlinear relationships could be examined without making any assumptions about specific forms of the dose response. The mechanisms by which these OCPs may affect sexual maturation are poorly understood; obtaining reproductive hormones in this cohort would provide insight into the underlying mechanisms. Additionally, although the onset of spermatogenesis (spermarche) was not a focus of this analysis, spermarche closely reflects the achievement of testis function during male puberty (Kulin et al. 1989; Schaefer et al. 1990). Obtaining data on spermaturia may contribute to a better understanding of the relationship between OCPs and sexual maturity because it may better predict the clinical stage of puberty (Schaefer et al. 1990).

The strengths of our study include a prospective design that followed a cohort of prepubertal boys to sexual maturity, using three established pubertal measures, including a highly precise method of testicular volume determination, in a population with a range of OCP serum concentrations. Additionally, the retention rate was high, and there was minimal differential loss to follow-up by demographic factors. Finally, one physician conducted all pubertal assessments across the nine annual physical exam visits, thus eliminating interexaminer variability (Carlsen et al. 2000).

## Conclusion

Our novel findings add new evidence to the limited literature that suggests that prepubertal exposure to environmental OCPs at relatively high levels, specifically HCB and βHCH, may affect age at sexual maturity in boys. Additional research is warranted to understand the implications of environmentally induced shifts in age at pubertal onset and sexual maturity on reproductive as well as psychosocial health.

## Supplemental Material

(395 KB) PDF
