# Supplementary material for: Prepubertal Serum Concentrations of Organochlorine Pesticides and Age at Sexual Maturity in Russian Boys
Source: Environ Health Perspect. 2015 May 22;123(11):1216–21. doi: 10.1289/ehp.1409022 (PMC4629743; doi:10.1289/ehp.1409022)
Supplement: (395 KB) PDF [file ehp.1409022.s001.acco.pdf]

Environ Health Perspect
DOI: 10.1289/ehp.1409022

Note to Readers: *EHP* strives to ensure that all journal content is accessible to all readers. However, some figures and Supplemental Material published in *EHP* articles may not conform to 508 standards due to the complexity of the information being presented. If you need assistance accessing journal content, please contact ehp508@niehs.nih.gov. Our staff will work with you to assess and meet your accessibility needs within 3 working days.

Supplemental Material

Prepubertal Serum Concentrations of Organochlorine Pesticides and Age at Sexual Maturity in Russian Boys

Thuy Lam, Paige L. Williams, Mary M. Lee, Susan A. Korrick, Linda S. Birnbaum, Jane S. Burns, Oleg Sergeyev, Boris Revich, Larisa M. Altshul, Donald G. Patterson Jr., and Russ Hauser

Table of Contents

Table S1. Adjusted Mean Shifts in Age at Sexual Maturity (Months, 95% CIs) by Quartiles of Wet-Weight Serum OCPs Additionally Adjusted for Maternal Age at Menarche and Baseline Height Z-scores and BMI in 350 Russian Boys

Table S2. Adjusted Mean Shifts in Age at Sexual Maturity (Months, 95% CIs) Comparing Models of Wet-Weight versus Lipid-Normalized OCP Measures in 350 Russian Boys

Table S3. Adjusted Mean Shifts in Age at Sexual Maturity (Months, 95% CIs) by Quartiles of Lipid-Normalized Serum OCPs Additionally Adjusted for Maternal Age at Menarche and Baseline Height Z-scores and BMI in 350 Russian Boys

Table S4. Adjusted Mean Shifts in Age at Sexual Maturity (Months, 95% CIs) by Quartiles of Wet-Weight Serum OCP Concentrations Adjusted for Serum Concentrations of Other OCPs in 350 Russian Boys

Figure S1. Adjusted Mean Shifts in Age at Sexual Maturity (Months, 95% CIs) by Quartiles of Lipid-Normalized Serum OCP concentrations in 350 Russian Boys

Table S1. Adjusted Mean Shifts in Age at Sexual Maturity (Months, 95% CIs) by Quartiles of Wet-Weight Serum OCPs Additionally Adjusted for Maternal Age at Menarche and Baseline Height Z-scores and BMI in 350 Russian Boys.

	G5		TV ≥ 20 mL		P5	
	Sensitivity Analysis: Maternal age at Menarche[a] (n=322)	Sensitivity Analysis: BMI + Height[b] (n=347)	Sensitivity Analysis: Maternal age at Menarche[c] (n=322)	Sensitivity Analysis: BMI + Height[d] (n=347)	Sensitivity Analysis: Maternal age at Menarche[e] (n=322)	Sensitivity Analysis: BMI + Height[f] (n=347)
**Serum OCP Quartile**	**Mean Shift (mos) and 95% CI**	**Mean Shift (mos) and 95% CI**	**Mean Shift (mos) and 95% CI**	**Mean Shift (mos) and 95% CI**	**Mean Shift (mos) and 95% CI**	**Mean Shift (mos) and 95% CI**
**HCB[g]**						
Q1 (low)	Reference	Reference	Reference	Reference	Reference	Reference
Q2	0.74 (-4.60, 6.08)	2.84 (-2.21, 7.90)	1.72 (-3.06, 6.50)	3.15 (-1.40, 1.84)	2.68 (-2.98, 8.33)	3.23 (-2.07, 8.53)
Q3	4.14 (-1.26, 9.54)	4.97 (-0.26, 10.20)	4.38 (-0.43, 9.18)	4.47 (-0.26, 3.43)	9.41 (3.49, 15.33)	7.27 (1.55, 12.99)
Q4 (high)	2.96 (-2.43, 8.35)	3.23 (-2.00, 8.45)	4.38 (-0.49, 9.24)	4.48 (-0.26, 3.43)	7.65 (1.75, 13.55)	6.17 (0.46, 11.88)
Trend p	0.16	0.18	0.04	0.06	0.002	0.02
**β-HCH[h]**						
Q1 (low)	Reference	Reference	Reference	Reference	Reference	Reference
Q2	1.50 (-3.79, 6.79)	3.34 (-1.76, 8.45)	0.10 (-4.66, 4.86)	0.92 (-3.70, 5.54)	0.98 (-4.73, 6.70)	-0.46 (-5.90, 4.99)
Q3	4.80 (-0.65, 10.24)	6.41 (1.01, 11.82)	3.16 (-1.75, 8.07)	3.60 (-1.39, 8.59)	6.76 (0.72, 12.80)	3.85 (-2.18, 9.89)
Q4 (high)	2.64 (-2.82, 8.10)	2.93 (-2.59, 8.45)	3.21 (-1.73, 8.16)	2.55 (-2.52, 7.61)	4.11 (-1.94, 10.17)	-0.22 (-6.38, 5.94)
Trend p	0.20	0.22	0.11	0.24	0.06	0.75
***p,p'*-DDE[i]**						
Q1 (low)	Reference	Reference	Reference	Reference	Reference	Reference
Q2	-0.42 (-5.79, 4.95)	-0.90 (-6.23, 4.42)	-0.21 (-5.06, 4.63)	0.24 (-4.55, 5.03)	-0.79 (-6.66, 5.08)	-3.34 (-9.03, 2.35)
Q3	0.14 (-5.28, 5.55)	-1.35 (-6.72, 4.01)	1.10 (-3.75, 5.95)	-0.30 (-5.16, 4.56)	1.74 (-4.18, 7.66)	-2.74 (-8.57, 3.09)
Q4 (high)	2.57 (-3.01, 8.15)	-0.02 (-5.69, 5.65)	1.58 (-3.49, 6.66)	-0.17 (-5.33, 5.00)	4.50 (-1.61, 10.62)	-1.12 (-7.24, 5.01)
Trend p	0.36	0.96	0.45	0.89	0.10	0.82

G5: Tanner stage 5 for genitalia growth; P5: Tanner stage 5 for pubic hair growth

[a]G5 sensitivity model adjusted for baseline covariates: boys' total serum lipids, macronutrients (total caloric intake, percent calories from dietary carbohydrates, fat; and protein), maternal age at menarche; missing maternal age at menarche, n=26, macronutrients, n=3. [b]G5 sensitivity model adjusted for baseline covariates: boys' total serum lipids, macronutrients (total caloric intake, percent calories from dietary carbohydrates, fat, and protein), WHO BMI categories, WHO height z-scores; missing macronutrients, n=3. [c]TV ≥ 20 mL sensitivity model adjusted for baseline covariates: boys' total serum lipids, birth weight, blood lead levels, biological father's absence from the household, maternal age at menarche; missing birth weight, n=1, maternal age

at menarche, n=26. [d]TV ≥ 20 mL sensitivity model adjusted for baseline covariates: boys' total serum lipids, birth weight, blood lead levels, biological father's absence from the household; WHO BMI categories, and WHO height z-scores; missing birth weight, n=1. [e]P5 sensitivity model adjusted for baseline covariates: boys' total serum lipids, biological father's absence from the household, maternal age at menarche; missing maternal age at menarche, n=26. [f]P5 sensitivity model adjusted for baseline covariates: boys' total serum lipids, biological father's absence from the household, WHO BMI categories, and WHO height z-scores. [g]HCB wet-weight quartiles (Q1-Q4, pg/g serum): Q1, 169-516; Q2, 517-751; Q3, 752-1,156; Q4, 1,157-15,482. [h]β-HCH wet-weight quartiles (Q1-Q4, pg/g serum): Q1, 209-567; Q2, 568-814; Q3, 815-1,294; Q4, 1,295-13,732. [i]*p,p*'-DDE wet-weight quartiles (Q1-Q4, pg/g serum): Q1, 261-907; Q2, 908-1,406; Q3, 1,407-2,327, Q4, 2,328-41,301.

Table S2. Adjusted Mean Shifts in Age at Sexual Maturity (Months, 95% CIs) Comparing Models of Wet-Weight versus Lipid-Normalized OCP. Measures in 350 Russian Boys.

	G5 (n=347)		TV > 20 mL (n=349)		P5 (n=350)	
	Wet-Weight model[a]	Lipid-Normalized model[b]	Wet-Weight model[c]	Lipid-Normalized model[d]	Wet-Weight model[e]	Lipid-Normalized model[f]
**Serum OCP Quartile**	**Mean Shift (mos) and 95% CI**	**Mean Shift (mos) and 95% CI**	**Mean Shift (mos) and 95% CI**	**Mean Shift (mos) and 95% CI**	**Mean Shift (mos) and 95% CI**	**Mean Shift (mos) and 95% CI**
**HCB[g,h]**						
Q1 (low)	Reference	Reference	Reference	Reference	Reference	Reference
Q2	2.78 (-2.53, 8.09)	2.41 (-2.89, 7.71)	3.05 (-1.72, 7.81)	-0.73 (-5.54, 4.08)	4.43 (-1.28, 10.14)	6.08 (0.22, 11.94)
Q3	5.64 (0.34, 10.94)	4.62 (-0.68, 9.92)	5.34 (0.57, 10.10)	3.70 (-1.10, 8.49)	11.20 (5.27, 17.13)	7.85 (1.99, 13.72)
Q4 (high)	3.71 (-1.59, 9.00)	3.43 (-1.91, 8.77)	5.01 (0.21, 9.82)	2.81 (-2.04, 7.66)	9.73 (3.78, 15.67)	9.02 (2.95, 15.08)
Trend p	0.10	0.14	0.02	0.10	<0.001	0.003
**β-HCH[i,j]**						
Q1 (low)	Reference	Reference	Reference	Reference	Reference	Reference
Q2	2.18 (-3.12, 7.48)	6.44 (1.12, 11.75)	0.10 (-4.68, 4.88)	4.43 (-0.33, 9.19)	1.17 (-4.63, 6.96)	6.14 (0.32, 11.96)
Q3	5.69 (0.36, 11.02)	4.97 (-0.31, 10.24)	3.71 (-1.13, 8.54)	3.95 (-0.84, 8.73)	8.67 (2.61, 14.74)	9.33 (3.42, 15.23)
Q4 (high)	3.71 (-1.65, 9.08)	5.68 (0.36, 10.99)	3.63 (-1.27, 8.53)	5.60 (0.72, 10.48)	5.99 (-0.08, 12.07)	7.44 (11.45, 13.42)
Trend p	0.09	0.07	0.07	0.04	0.01	0.01
***p,p*'-DDE[k,l]**						
Q1 (low)	Reference	Reference	Reference	Reference	Reference	Reference
Q2	-1.68 (-6.98, 3.61)	0.65 (-4.65, 5.95)	-0.32 (-5.10, 4.47)	-0.54 (-5.43, 4.35)	-0.30 (-6.19, 5.59)	-0.11 (-6.15, 5.94)
Q3	-1.34 (-6.67, 3.99)	0.61 (-4.65, 5.88)	-0.09 (-4.89, 4.71)	0.02 (-4.77, 4.81)	1.67 (-4.29, 7.63)	2.42 (-3.54, 8.38)
Q4 (high)	2.52 (-2.92, 7.97)	4.89 (-0.51, 10.30)	2.45 (-2.49, 7.39)	3.32 (-1.61, 8.24)	6.19 (0.11, 12.27)	5.67 (-0.40, 11.74)
Trend p	0.37	0.10	0.35	0.19	0.04	0.05

G5: Tanner stage 5 for genitalia growth; P5: Tanner stage 5 for pubic hair growth.

[a]G5 wet-weight model adjusted for baseline covariates: boys' total serum lipids, macronutrients (total caloric intake, percent calories from dietary carbohydrates, fat, and protein); Missing macronutrients, n=3. [b]G5 lipid-normalized model adjusted for baseline covariates: boys' macronutrients (total caloric intake, percent calories from dietary carbohydrates, fat, and protein); Missing macronutrients, n=3. [c]TV ≥ 20 mL wet-weight model adjusted for baseline covariates: boys' total serum lipids, birth weight, blood lead levels, biological father's absence from the household; Missing birth weight, n=1. [d]TV ≥ 20 mL lipid-normalized model adjusted for baseline covariates: boys' birth weight, blood lead levels, biological father's absence from the household; Missing birth weight, n=1. [e]P5 wet-weight model adjusted for baseline covariates: boys' total serum lipids, biological father's absence from the household. [f]P5 lipid-normalized model adjusted for baseline

covariates: boys' biological father's absence from the household. [g]HCB wet-weight quartiles (Q1-Q4, pg/g serum): Q1, 169-516; Q2, 517-751; Q3, 752-1,156; Q4, 1,157-15,482. [h]HCB lipid-normalized quartiles (Q1-Q4, ng/g lipid): Q1, 31-105; Q2, 106-158; Q3, 159-246; Q4, 247-2,660. [i]β-HCH wet-weight quartiles (Q1-Q4, pg/g serum): Q1, 209-567; Q2, 568-814; Q3, 815-1,294; Q4, 1,295-13,732. [j]β-HCH lipid-normalized quartiles (Q1-Q4, ng/g lipid): Q1, 39-115; Q2, 116-168; Q3, 169-269; Q4, 270-2,860. [k]*p,p*'-DDE wet-weight quartiles (Q1-Q4, pg/g serum): Q1, 261-907; Q2, 908-1406; Q3, 1,407-2,327, Q4, 2,328-41,301. [l]*p,p*'-DDE lipid-normalized quartiles (Q1-Q4, ng/g lipid): Q1, 48-189; Q2, 190-292; Q3, 293-493, Q4, 494-9,370.

Table S3. Adjusted Mean Shifts in Age at Sexual Maturity (Months, 95% CIs) by Quartiles of Lipid-Normalized Serum OCPs Additionally Adjusted for Maternal Age at Menarche and Baseline Height Z-scores and BMI in 350 Russian Boys.

	G5		TV ≥ 20 mL		P5	
	**Sensitivity Analysis: Maternal age at Menarche[a] (n=322)**	**Sensitivity Analysis: BMI + Height[b] (n=347)**	**Sensitivity Analysis: Maternal age at Menarche[c] (n=324)**	**Sensitivity Analysis: BMI + Height[d] (n=349)**	**Sensitivity Analysis: Maternal age at Menarche[e] (n=324)**	**Sensitivity Analysis: BMI + Height[f] (n=350)**
**Serum OCP Quartile**	**Mean Shift (mos) and 95% CI**	**Mean Shift (mos) and 95% CI**	**Mean Shift (mos) and 95% CI**	**Mean Shift (mos) and 95% CI**	**Mean Shift (mos) and 95% CI**	**Mean Shift (mos) and 95% CI**
**HCB[g]**						
Q1 (low)	Reference	Reference	Reference	Reference	Reference	Reference
Q2	1.17 (-4.17, 6.51)	1.66 (-3.47, 6.80)	-1.27 (-6.08, 3.54)	-1.45 (-6.10, 3.20)	4.84 (-0.95, 10.63)	3.09 (-2.40, 8.58)
Q3	3.12 (-2.27, 8.51)	4.03 (-1.29, 9.35)	3.18 (-1.65, 8.01)	2.59 (-2.23, 7.42)	6.77 (0.95, 12.59)	3.75 (-1.93, 9.44)
Q4 (high)	2.81 (-2.67, 8.29)	2.85 (-2.46, 8.16)	2.40 (-2.54, 7.33)	2.05 (-2.76, 6.87)	7.03 (0.99, 13.07)	4.90 (-0.92, 10.73)
Trend p	0.23	0.22	0.13	0.19	0.01	0.10
**β-HCH[h]**						
Q1 (low)	Reference	Reference	Reference	Reference	Reference	Reference
Q2	6.92 (1.65, 12.19)	7.48 (2.33, 12.63)	4.84 (0.12, 9.56)	5.04 (0.40, 9.68)	5.83 (0.11, 11.54)	4.11 (-1.38, 9.59)
Q3	4.97 (-0.32, 10.27)	6.07 (0.74, 11.40)	4.49 (-0.29, 9.27)	4.23 (-0.68, 9.14)	8.49 (2.68, 14.30)	5.18 (-0.66, 11.02)
Q4 (high)	5.10 (-0.27, 10.48)	4.80 (-0.69, 10.30)	5.34 (0.43, 10.25)	4.42 (-0.63, 9.47)	5.10 (-0.83, 11.04)	1.54 (-4.51, 7.58)
Trend p	0.12	0.18	0.04	0.14	0.04	0.56
***p,p*'-DDE[i]**						
Q1 (low)	Reference	Reference	Reference	Reference	Reference	Reference
Q2	1.42 (-3.97, 6.82)	1.14 (-4.14, 6.41)	-0.55 (-5.48, 4.37)	-0.68 (-5.50, 4.14)	-0.15 (-6.14, 5.84)	-2.94 (-8.71, 2.82)
Q3	1.75 (-3.56, 7.06)	0.56 (-4.72, 5.84)	1.06 (-3.75, 5.87)	-0.40 (-5.23, 4.42)	2.73 (-3.15, 8.61)	-1.43 (-7.25, 4.38)
Q4 (high)	4.57 (-0.92, 10.06)	2.38 (-3.22, 7.97)	2.27 (-2.72, 7.26)	0.17 (-4.94, 5.29)	4.06 (-2.00, 10.12)	-1.56 (-7.66, 4.54)
Trend p	0.12	0.48	0.30	0.92	0.12	0.77

G5: Tanner stage 5 for genitalia growth; P5: Tanner stage 5 for pubic hair growth.

[a]G5 sensitivity model adjusted for baseline covariates: boys' macronutrients (total caloric intake, percent calories from dietary carbohydrates, fat; and protein), maternal age at menarche; missing maternal age at menarche, n=26, macronutrients, n=3. [b]G5 sensitivity model adjusted for baseline covariates: boys' macronutrients (total caloric intake, percent calories from dietary carbohydrates, fat, and protein), WHO BMI categories, WHO

height z-scores; missing macronutrients, n=3. [c]TV ≥ 20 mL sensitivity model adjusted for baseline covariates: boys' birth weight, blood lead levels, biological father's absence from the household, maternal age at menarche; missing birth weight, n=1, maternal age at menarche, n=26. [d]TV ≥ 20 mL lipid-normalized model adjusted for baseline covariates boys' birth weight, blood lead levels, biological father's absence from the household, WHO BMI categories, WHO height z-scores; missing birth weight, n=1, maternal age at menarche, n=26. [e]P5 sensitivity model adjusted for baseline covariates: boys' biological father's absence from the household, maternal age at menarche; missing maternal age at menarche, n=26. [f]P5 sensitivity model adjusted for baseline covariates: biological father's absence from the household, WHO BMI categories, and WHO height z-scores. [g]HCB lipid-normalized quartiles (Q1-Q4, ng/g lipid): Q1, 31-105; Q2, 106-158; Q3, 159-246; Q4, 247-2,660
[h]β-HCH lipid-normalized quartiles (Q1-Q4, ng/g lipid): Q1, 39-115; Q2, 116-168; Q3, 169-269; Q4, 270-2,860. [i]*p,p*'-DDE lipid-normalized quartiles (Q1-Q4, ng/g lipid): Q1, 48-189; Q2, 190-292; Q3, 293-493, Q4, 494-9,370.

Table S4. Adjusted Mean Shifts in Age at Sexual Maturity (Months, 95% CIs) by Quartiles of Serum OCP Wet-Weight Concentrations Adjusted for Serum Concentrations of Other OCPs in 350 Russian Boys.

Serum OCP Quartile	G5 (n=347)[a]		TV ≥ 20 mL (n=349)[b]		P5 (n=350)[c]	
	Mean Shift (months) and 95% CI	P-value	Mean Shift (months) and 95% CI	P-value	Mean Shift (months) and 95% CI	P-value
**HCB and β-HCH in model[d,e]**						
HCB Q1 (low)	Reference		Reference		Reference	
HCB Q2	2.09 (-3.31, 7.49)	0.45	2.60 (-2.28, 7.48)	0.30	3.76 (-2.03, 9.55)	0.20
HCB Q3	4.16 (-1.81, 10.13)	0.17	4.16 (-1.25, 9.58)	0.13	9.51 (2.88, 16.14)	0.005
HCB Q4 (high)	2.57 (-3.54, 8.69)	0.41	3.90 (-1.67, 9.47)	0.17	8.85 (2.08, 15.62)	0.01
HCB P for trend		0.37		0.13		0.004
β-HCH Q1 (low)	Reference		Reference		Reference	
β-HCH Q2	2.03 (-3.26, 7.33)	0.45	-0.14 (2.44, -4.92)	0.95	1.10 (-4.62, 6.82)	0.71
β-HCH Q3	4.15 (-1.64, 9.94)	0.16	1.99 (-3.27, 7.24)	0.46	4.96 (-1.45, 11.36)	0.13
β-HCH Q4 (high)	2.19 (-3.92, 8.30)	0.48	1.57 (-4.02, 7.15)	0.58	1.14 (-5.62, 7.91)	0.74
β-HCH P for trend		0.33		0.43		0.40
**HCB and *p,p'*-DDE in model[d,f]**						
HCB Q1 (low)	Reference		Reference		Reference	
HCB Q2	3.08 (2.73, -2.27)	0.26	3.20 (-1.62, 8.01)	0.19	4.47 (-1.27, 10.20)	0.13
HCB Q3	5.65 (0.28, 11.02)	0.04	5.31 (0.45, 10.17)	0.03	10.71 (4.72, 16.70)	<0.001
HCB Q4 (high)	3.97 (-1.53, 9.46)	0.16	5.14 (0.13, 10.14)	0.04	9.40 (3.24, 15.56)	0.003
HCB P for trend		0.14		0.04		0.001
p,p'-DDE Q1 (low)	Reference		Reference		Reference	
p,p'-DDE Q2	-2.34 (-7.71, 3.03)	0.39	-1.26 (-6.11, 3.58)	0.61	-1.93 (-7.82, 3.96)	0.52
p,p'-DDE Q3	-2.46 (-7.92, 3.00)	0.38	-1.46 (-6.37, 3.44)	0.56	-0.91 (-6.94, 5.12)	0.77
p,p'-DDE Q4 (high)	1.34 (-4.25, 6.94)	0.64	0.91 (-4.16, 5.98)	0.73	3.27 (-2.88, 9.43)	0.30
p,p'-DDE *P for trend*		0.62		0.72		0.26
**β-HCH and *p,p'*-DDE in model[e,f]**						
β-HCH Q1 (low)	Reference		Reference		Reference	
β-HCH Q2	2.68 (-2.68, 8.04)	0.33	0.34 (-4.51, 5.19)	0.89	1.17 (-4.70, 7.04)	0.70
β-HCH Q3	6.24 (0.54, 11.95)	0.03	4.05 (-1.15, 9.25)	0.13	8.10 (1.59, 14.61)	0.01
β-HCH Q4 (high)	3.62 (-2.54, 9.78)	0.25	3.72 (-1.3, 9.36)	0.20	4.38 (-2.74, 11.51)	0.23
β-HCH P for trend		0.15		0.11		0.08

Serum OCP Quartile	G5 (n=347)[a] Mean Shift (months) and 95% CI	P-value	TV ≥ 20 mL (n=349)[b] Mean Shift (months) and 95% CI	P-value	P5 (n=350)[c] Mean Shift (months) and 95% CI	P-value
p,p'-DDE Q1 (low)	Reference		Reference		Reference	
p,p'-DDE Q2	-2.65 (-8.00, 2.70)	0.33	-1.08 (-5.93, 3.78)	0.66	-1.40 (-7.31, 4.51)	0.64
p,p'-DDE Q3	-3.36 (-9.10, 2.37)	0.25	-1.81 (-6.98, 3.35)	0.49	-1.00 (-7.42, 5.41)	0.76
p,p'-DDE Q4 (high)	0.44 (-5.80, 6.68)	0.89	0.07 (-5.61, 5.76)	0.98	2.98 (-4.06, 10.01)	0.41
p,p'-DDE *P for trend*		0.99		0.96		0.45
**HCB, β-HCH and *p,p*'-DDE in model[d,e,f]**						
HCB Q1 (low)	Reference		Reference		Reference	
HCB Q2	2.56 (-2.84, 7.95)	0.35	2.83 (-2.06, 7.73)	0.26	4.23 (-1.56, 10.01)	0.15
HCB Q3	4.53 (-1.43, 10.48)	0.14	4.33 (-1.09, 9.75)	0.12	10.04 (3.42, 16.65)	0.003
HCB Q4 (high)	3.46 (-2.72, 9.64)	0.27	4.34 (-1.30, 9.98)	0.13	9.93 (3.05, 16.80)	0.005
HCB P for trend		0.37		0.13		0.004
β-HCH Q1 (low)	Reference		Reference		Reference	
β-HCH Q2	2.51 (-2.83, 7.86)	0.36	0.12 (-4.72, 4.96)	0.96	1.14 (-4.64, 6.92)	0.70
β-HCH Q3	4.48 (-1.68, 10.63)	0.15	2.24 (-3.36, 7.84)	0.43	4.02 (-2.82, 10.87)	0.25
β-HCH Q4 (high)	1.62 (-5.26, 8.51)	0.64	1.40 (-4.90, 7.70)	0.66	-1.34 (-9.15, 6.47)	0.74
β-HCH P for trend		0.40		0.48		0.75
p,p'-DDE Q1 (low)	Reference		Reference		Reference	
p,p'-DDE Q2	-2.89 (-8.29, 2.52)	0.30	-1.50 (-6.40, 3.40)	0.55	-2.44 (-8.36, 3.48)	0.42
p,p'-DDE Q3	-3.48 (-9.22, 2.26)	0.24	-2.05 (-7.22, 3.12)	0.44	-1.39 (-7.76, 4.99)	0.67
p,p'-DDE Q4 (high)	0.63 (-5.60, 6.87)	0.84	0.16 (-5.51, 5.83)	0.96	3.45 (-3.52, 10.42)	0.33
p,p'-DDE *P for trend*		0.97		0.98		0.42

G5: Tanner stage 5 for genitalia growth; P5: Tanner stage 5 for pubic hair growth.

[a]G5 model adjusted for baseline covariates: boys' total serum lipids, macronutrients (total caloric intake, percent calories from dietary carbohydrates, fat; and protein); missing macronutrients, n=3. [b]TV ≥ 20 mL model adjusted for baseline covariates: boys' total serum lipids, birth weight, blood lead levels, biological father's absence from the household; missing birth weight, n=1. [c]P5 model adjusted for baseline covariates: boys' total serum lipids, biological father's absence from the household. [d]HCB wet-weight quartiles (Q1-Q4, pg/g serum): Q1, 169-516; Q2, 517-751; Q3, 752-1,156; Q4, 1,157-15,482. [e]β-HCH wet-weight quartiles (Q1-Q4, pg/g serum): Q1, 209-567; Q2, 568-814; Q3, 815-1,294; Q4, 1,295-13,732. [f]*p,p*'-DDE wet-weight quartiles (Q1-Q4, pg/g serum): Q1, 261-907; Q2, 908-1,406; Q3, 1,407-2,327, Q4, 2,328-41,301.

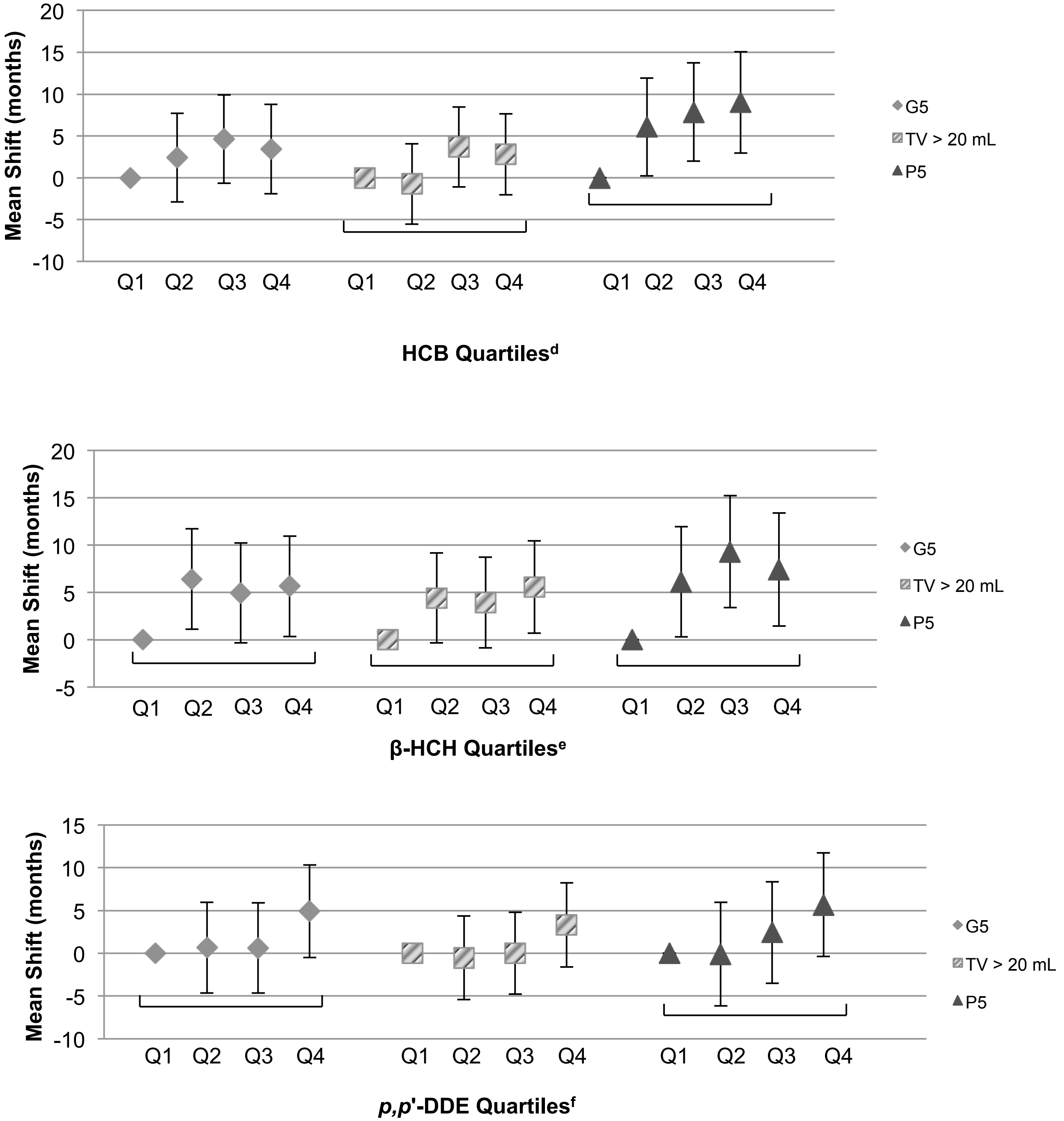

Figure S1. Adjusted Mean Shifts in Age at Sexual Maturity (Months, 95% CIs) by Quartiles of Lipid-Normalized Serum OCP concentrations in 350 Russian Boys.[a,b,c.]

*p ≤ 0.05. **p ≤ 0.10. Shift in months is relative to Q1 (reference). G5: Tanner stage 5 for genitalia growth; P5: Tanner stage 5 for pubic hair growth. [a]G5 model adjusted for baseline covariates: boys' macronutrients (total caloric intake, percent calories from dietary carbohydrates, fat, protein); missing macronutrients, n=3.

[b]TV ≥ 20 mL model adjusted for baseline covariates: boys' birth weight, blood lead levels, biological father's absence from the household; missing birth weight, n=1. [c]P5 model adjusted for baseline covariates: biological father's absence from the household. [d]HCB lipid-normalized quartiles (Q1-Q4, ng/g lipid): Q1, 31-105; Q2, 106-158; Q3, 159-246; Q4, 247-2,660. [e]β-HCH lipid-normalized quartiles (Q1-Q4, ng/g lipid): Q1, 39-115; Q2, 116-168; Q3, 169-269; Q4, 270-2,860. [f]*p,p*'-DDE lipid-normalized quartiles (Q1-Q4, ng/g lipid): Q1, 48-189; Q2, 190-292; Q3, 293-493, Q4, 494-9,370.
